# Supplementary material for: The methyltransferase N6AMT1 participates in the cell cycle by regulating cyclin E levels
Source: PLoS One. 2024 Feb 23;19(2):e0298884. doi: 10.1371/journal.pone.0298884 (PMC10889616; doi:10.1371/journal.pone.0298884)
Supplement: S1 Fig — (A) The proliferation rate of the U2OS, ΔN6AMT1#1 and ΔN6AMT1#2 cell lines were determined using an xCELLigence Real-Time cell analyser, and data were recorded for a total of 7 days. (B) qRT‒PCR verification of cyclin A, cyclin B and cyclin E levels in HEK293ΔN6AMT1 cells. The average of two biologically independent samples is shown. Statistical analysis was performed with GraphPad Prism 8.4.3. (C) Unsynchronized cells and cells that were synchronized with serum depletion and thymidine treatment were analysed via Western blotting with antibodies against Cyclin A, Cyclin B1, Cyclin E, N6AMT1 and GAPDH. 10 μg of total protein was loaded per well. (D-F) Cyclin A, cyclin B and cyclin E levels in U2OS and ΔN6AMT1#1 cells. Western blotting images were quantified using ImageJ, and the average of three biologically independent samples is shown. Statistical analysis was performed with GraphPad Prism 8.4.3. The data are presented as the means ± SDs; *P < 0,05, **P < 0,001, ***P < 0,0001 according to multiple t tests. (PDF) [file pone.0298884.s001.pdf]

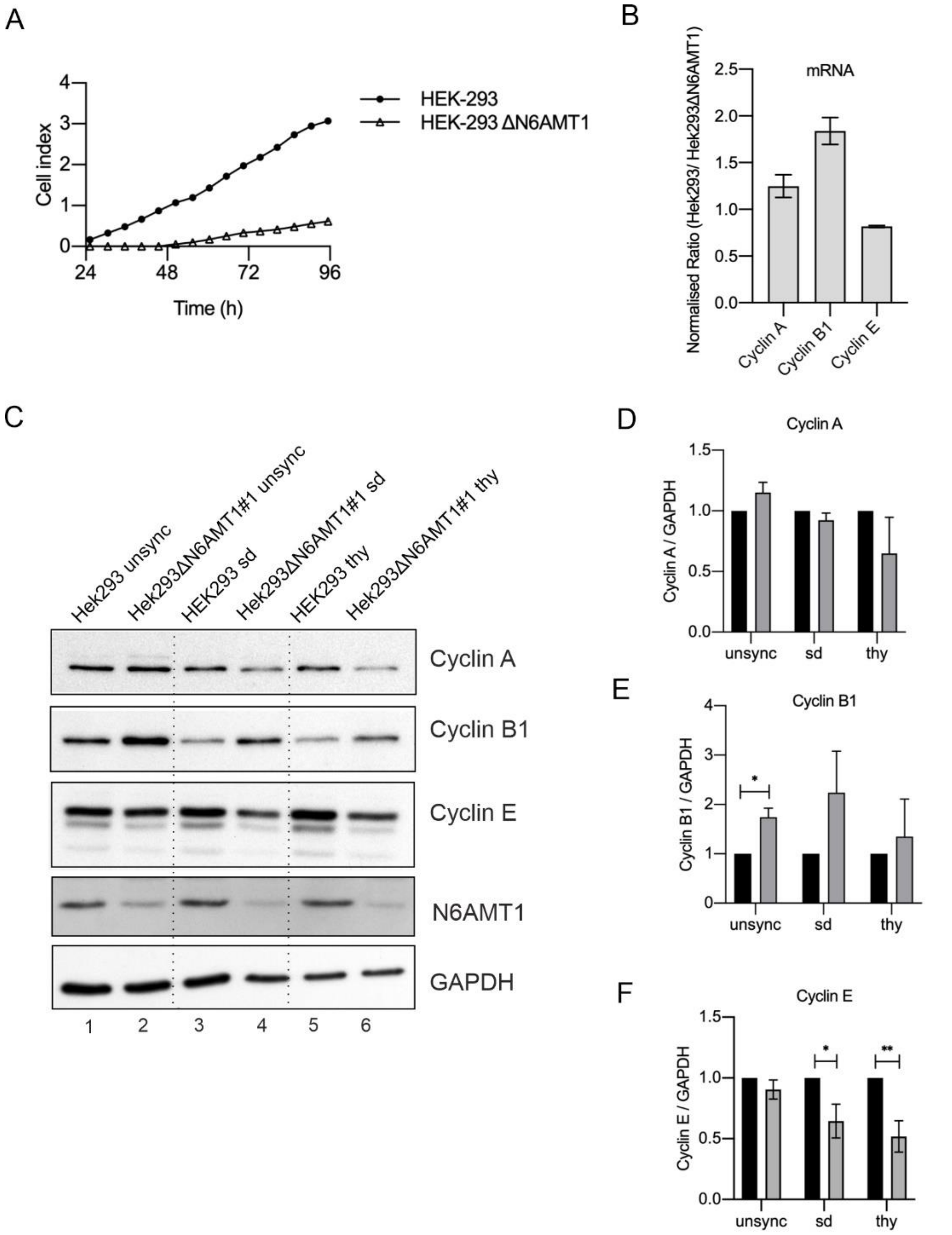

S1 Fig. **N6AMT is important for cell proliferation and cyclin E regulation in Hek293 cells**

(A) The proliferation rate of the U2OS, ΔN6AMT1#1 and ΔN6AMT1#2 cell lines were determined using an xCELLigence Real-Time cell analyser, and data were recorded for a total of 7 days. (B) qRT–PCR verification of cyclin A, cyclin B and cyclin E levels in Hek293ΔN6AMT1 cells. The average of two biologically independent samples is shown. Statistical analysis was performed with GraphPad Prism 8.4.3. (C) Unsynchronized cells and cells that were synchronized with serum depletion and thymidine treatment were analysed via Western blotting with antibodies against Cyclin A, Cyclin B1, Cyclin E, N6AMT1 and GAPDH. 10 μg of total protein was loaded per well. (D-F) Cyclin A, cyclin B and cyclin E levels in U2OS and ΔN6AMT1#1 cells. Western blotting images were quantified using ImageJ, and the average of three biologically independent samples is shown. Statistical analysis was performed with GraphPad Prism 8.4.3. The data are presented as the means ± SDs; *P < 0,05, **P < 0,001, ***P < 0,0001 according to multiple t tests.
